# Supplementary material for: Combined Inhibition of Polo-Like Kinase-1 and Wee1 as a New Therapeutic Strategy to Induce Apoptotic Cell Death in Neoplastic Mast Cells
Source: Cancers (Basel). 2022 Jan 31;14(3):738. doi: 10.3390/cancers14030738 (PMC8833529; doi:10.3390/cancers14030738)
Supplement: Supplementary file 1 [file cancers-14-00738-s001.zip › cancers-1529089-supplementary-File S1-done.pdf]

cancers

MDPI

Supplementary reports:

A dose-escalation experiment was performed to define the drug doses required to induce sub-lethal effects in HMC-1.1 and HMC-1.2 cell lines. For this purpose, each drug, alone or in combination, was added at scalar doses into the cell culture medium, starting from 0.25 to 1 μM. After 24 hours treatment cells were evaluated for Annexin V uptake and the percentage of residual living cells was used to calculate IC50 values by using a dedicated software (Compusyn).

Compusyn reports are shown below.

Data for Drug: MK1775 [μM] in HMC-1.2 cell line

Dose (μM)	Effect (% of living cells)
0.25	0.92
0.5	0.87
0.75	0.69
1.0	0.55

4 data points entered.

Data for Drug: volasertib [μM]

Dose (μM)	Effect (% of living cells)
0.25	0.85
0.5	0.68
0.75	0.51
1.0	0.43

4 data points entered.

Data for Drug: danusertib [μM]

Dose (μM)	Effect (% of living cells)
0.25	0.88
0.5	0.72
0.75	0.53
1.0	0.48

4 data points entered.

Data for Drug Combo: (MK1775+danusertib [1:1])

Dose (μM)	Effect (% of living cells)
0.25	0.62
0.5	0.54
0.75	0.27
1.0	0.13

4 data points entered.

Total Dose (μM)	Effect (% of living cells)	CI Value
0.5	0.62	0.65277
1.0	0.54	1.06118
1.5	0.27	0.76950
2.0	0.13	0.58012

Data for Drug Combo: (MK1775+volasertib [1:1])

Dose (μM)	Effect (% of living cells)
0.25	0.60
0.5	0.51
0.75	0.19
1.0	0.10

4 data points entered.

Total Dose (μM)	Effect (% of living cells)	CI Value
0.5	0.60	0.50261
1.0	0.51	1.00001
1.5	0.19	0.65120
2.0	0.10	0.42010

Data for Drug: MK1775 [μM] in HMC-1.1 cell line

Dose (μM)	Effect (% of living cells)
0.25	0.98
0.5	0.92
0.75	0.70
1.0	0.58

4 data points entered.

Data for Drug: volasertib [μM]

Dose (μM)	Effect (% of living cells)
0.25	0.91
0.5	0.72
0.75	0.61
1.0	0.48

4 data points entered.

Data for Drug: danusertib [μM]

Dose (μM)	Effect (% of living cells)
0.25	0.81
0.5	0.68
0.75	0.47
1.0	0.35

4 data points entered.

Data for Drug Combo: (MK1775+danusertib [1:1])

Dose (μM)	Effect (% of living cells)
0.25	0.57
0.5	0.49
0.75	0.21
1.0	0.09

4 data points entered.

Total Dose (μM)	Effect (% of living cells)	CI Value
0.5	0.57	0.61423
1.0	0.49	1.01253
1.5	0.21	0.82475
2.0	0.09	0.52140

Data for Drug Combo: (MK1775+volasertib [1:1])

Dose (μM)	Effect (% of living cells)
0.25	0.60
0.5	0.51
0.75	0.19
1.0	0.10

4 data points entered.

Total Dose (μM)	Effect (% of living cells)	CI Value
0.5	0.60	0.50261
1.0	0.51	1.00001
1.5	0.19	0.82456
2.0	0.10	0.42010
