# Supplementary material for: Combined Inhibition of Polo-Like Kinase-1 and Wee1 as a New Therapeutic Strategy to Induce Apoptotic Cell Death in Neoplastic Mast Cells
Source: Cancers (Basel). 2022 Jan 31;14(3):738. doi: 10.3390/cancers14030738 (PMC8833529; doi:10.3390/cancers14030738)
Supplement: Supplementary file 1 [file cancers-14-00738-s001.zip › cancers-1529089-supplementary-File S2 - done.pdf]

FIGURE 1 E

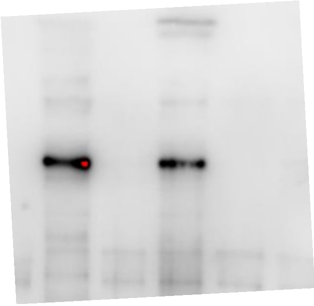

FIGURE 1 E LANE 1

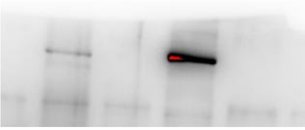

FIGURE 1 E LANE 2

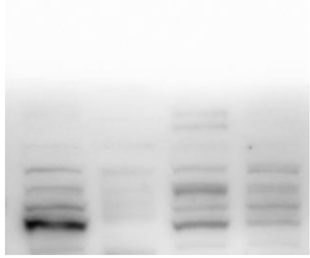

FIGURE 1 E LANE 3

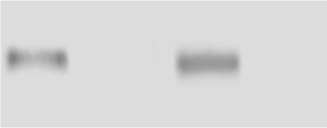

FIGURE 1 E LANE 4

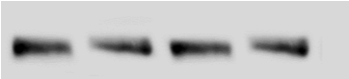

FIGURE 1 E LANE 5

FIGURE 1 F

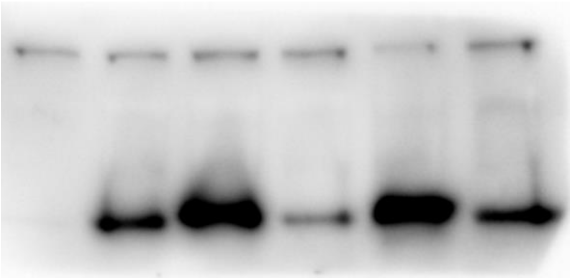

FIGURE 1 F LANE 1

FIGURE 1 F LANE 2

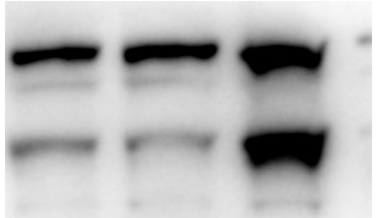

FIGURE 1 F HMC-1.1 LANE 3

FIGURE 1 F HMC-1.1 LANE 4

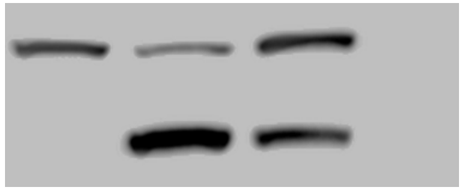

FIGURE 1 F HMC-1.2 LANE 3

FIGURE 1 F HMC-1.2 LANE 4

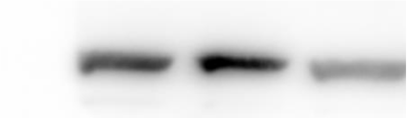

FIGURE 1 F HMC-1.1 LANE 5

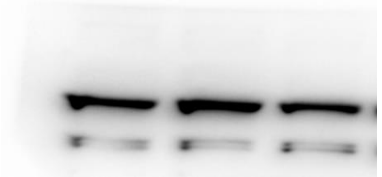

FIGURE 1 F HMC-1.2 LANE 5

FIGURE 2 B

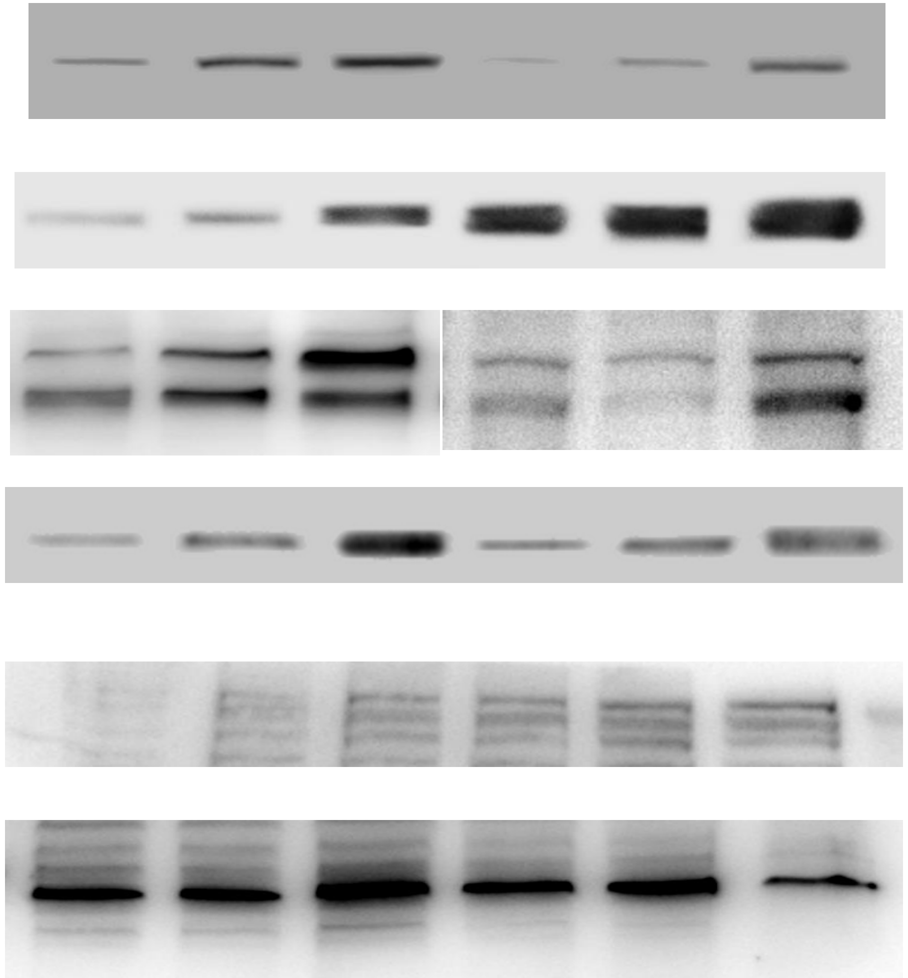

FIGURE 2 B LANE 1

FIGURE 2 B LANE 2

FIGURE 2 B LANE 3

FIGURE 2 B LANE 4

FIGURE 2 B LANE 5

FIGURE 2 B LANE 6

FIGURE 3 C

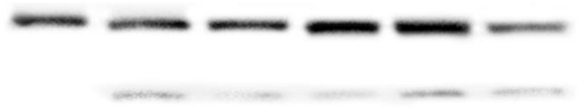

FIGURE 3 C LANE 5

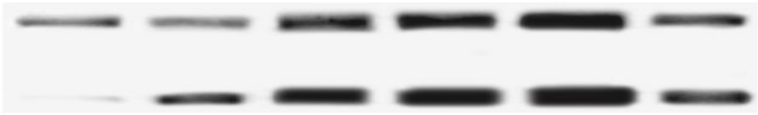

FIGURE 3 D LANE 5

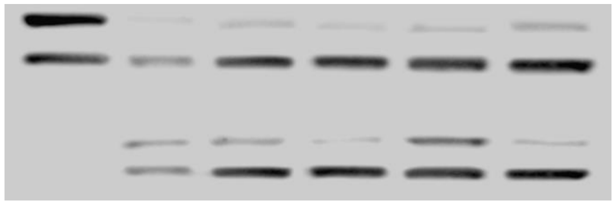

FIGURE 3 C LANE 4

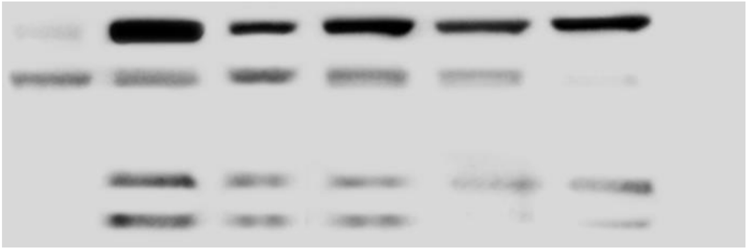

FIGURE 3 D LANE 4

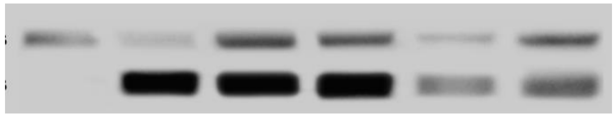

FIGURE 3 C LANE 6

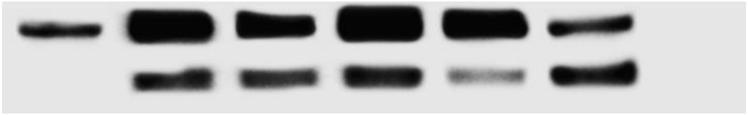

FIGURE 3 D LANE 6

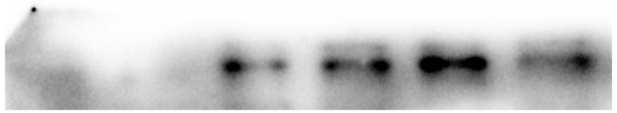

FIGURE 3 C LANE 7

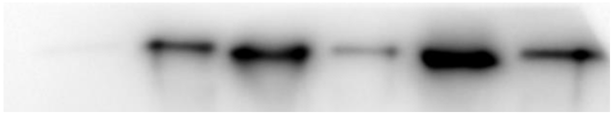

FIGURE 3 C LANE 8

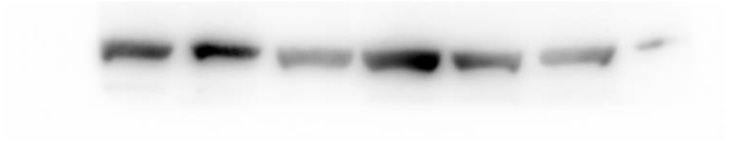

FIGURE 3 C LANE 9

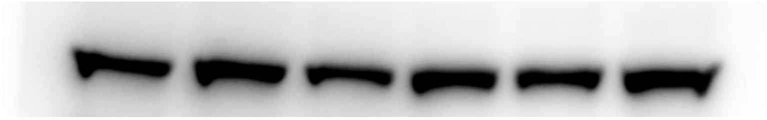

FIGURE 3 D LANE 9
