# Supplementary material for: Combined Inhibition of Polo-Like Kinase-1 and Wee1 as a New Therapeutic Strategy to Induce Apoptotic Cell Death in Neoplastic Mast Cells
Source: Cancers (Basel). 2022 Jan 31;14(3):738. doi: 10.3390/cancers14030738 (PMC8833529; doi:10.3390/cancers14030738)
Supplement: Supplementary file 1 [file cancers-14-00738-s001.zip › cancers-1529089-supplementary.pdf]

cancers MDPI

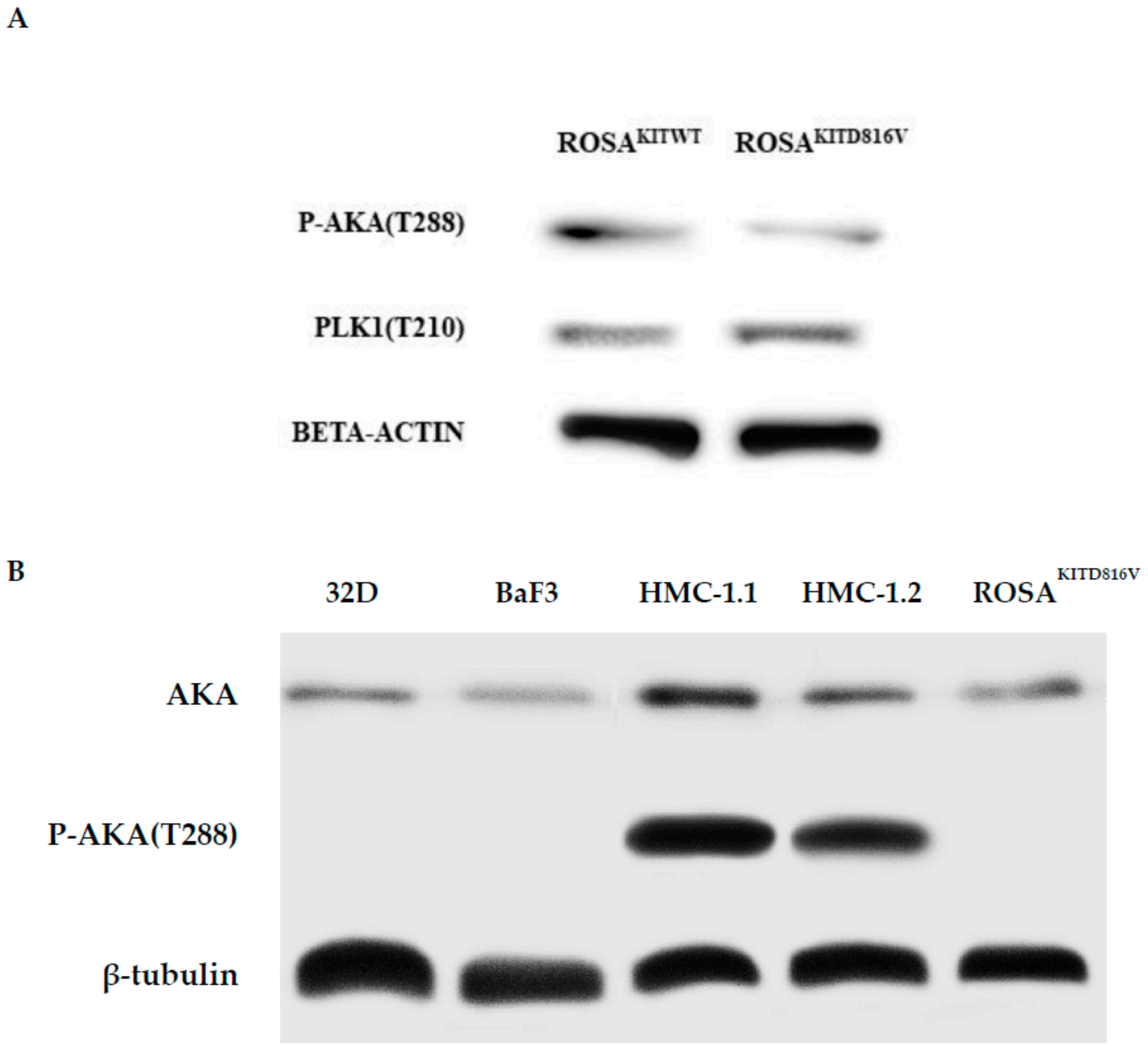

Figure S1. (**A**) AKA and Plk1 phosphorylation in SM cell lines: ROSA^{KITWT} and ROSA^{KITD816V}. (**B**) AKA expression and phosphorylation in normal cell lines (32D and BaF3) as compared to SM cell lines (HMC-1.1, HMC-1.2 and ROSA^{KITD816V}). Western blot analysis was performed to test AKA expression and activity.

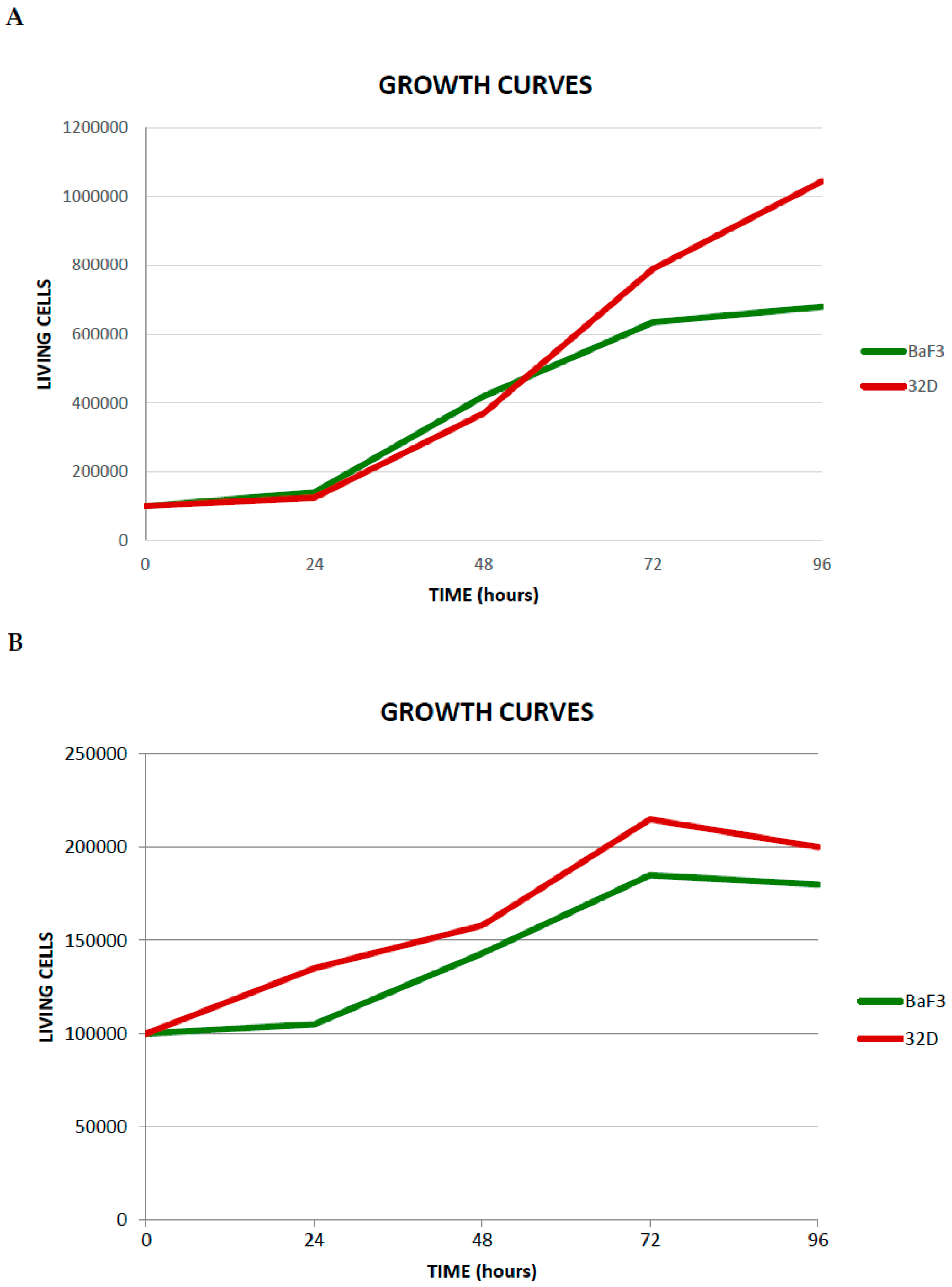
A
GROWTH CURVES
LIVING CELLS
1200000
1000000
800000
600000
400000
200000
0
0
24
48
72
96
TIME (hours)
BaF3
32D
B
GROWTH CURVES
LIVING CELLS
250000
200000
150000
100000
50000
0
0
24
48
72
96
TIME (hours)
BaF3
32D

C

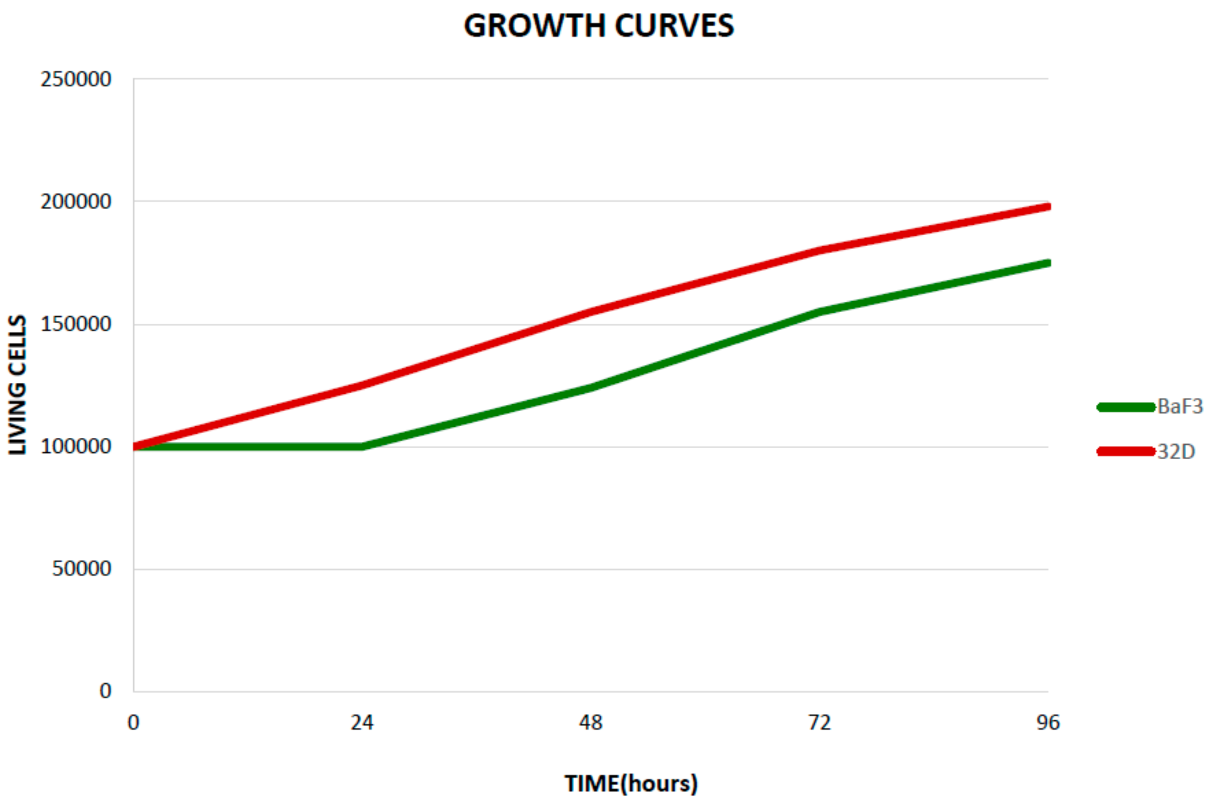

Figure S2. Effects of AKA or Plk1 inhibition in association with WEE1 inhibition in normal cells. To test the effects of the combination of danusertib and MK1775 or volasertib and MK1775 in BaF3 and 32D cell lines, a growth curve assay in liquid medium was performed. Cells were maintained under control conditions (**A**) or treated with danusertib or volasertib (100nM) + MK1775 (500 nM) for 24, 48, 72 and 96 hours (**B**) and (**C**) respectively). At each timepoint, cells were stained with trypan blue and counted by using a Burker chamber in order to discriminate living cells from dead cells and only the living cells were considered to perform the growth curves. As can be seen from Figure S2B,C, both the combination of danusertib and MK1775 and the combination of volasertib and MK1775 do not show any cytotoxic effects, although the growth of treated cells is obviously slower if compared with that of cells cultured in control medium (Figure S2A).

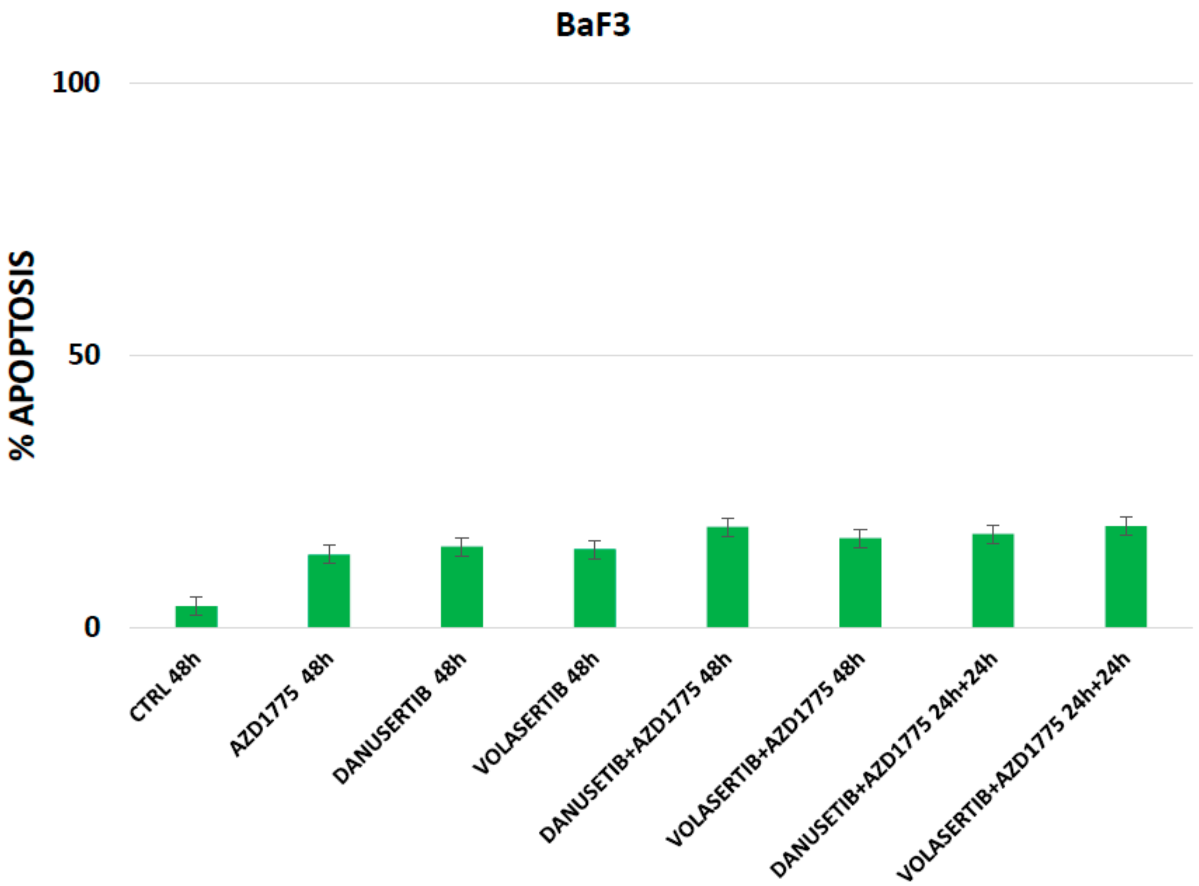
BaF3
100
50
0
% APOPTOSIS
CTRL 48h
AZD1775 48h
DANUSERTIB 48h
VOLASERTIB 48h
DANUSETIB+AZD1775 48h
VOLASERTIB+AZD1775 48h
DANUSETIB+AZD1775 24h+24h
VOLASERTIB+AZD1775 24h+24h

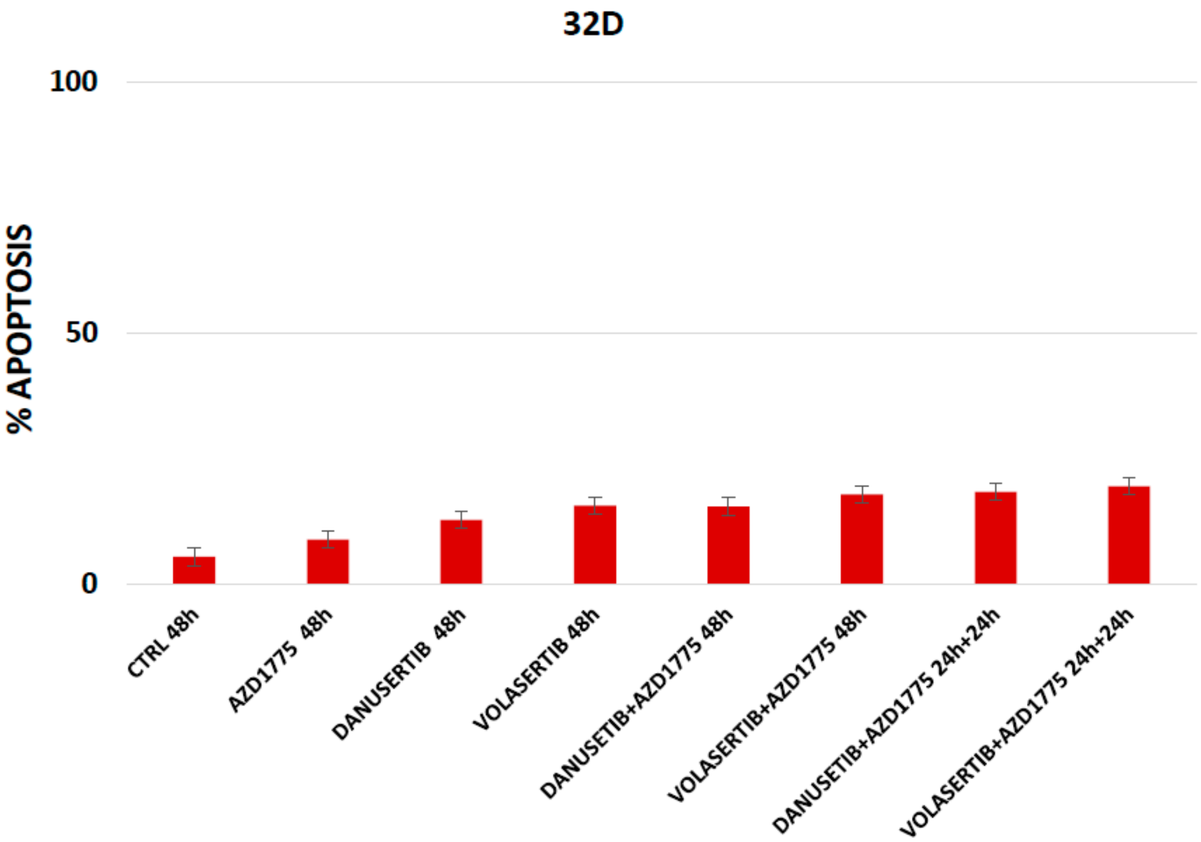
32D
100
50
0
% APOPTOSIS
CTRL 48h
AZD1775 48h
DANUSERTIB 48h
VOLASERTIB 48h
DANUSETIB+AZD1775 48h
VOLASERTIB+AZD1775 48h
DANUSETIB+AZD1775 24h+24h
VOLASERTIB+AZD1775 24h+24h

Figure S3. Effects of AKA or Plk1 inhibition in association with WEE1 inhibition. Flow cytometry analysis of apoptosis induction in BaF3 and 32D cells following 48h treatment with danusertib or volasertib alone or in combination with MK1775 (schedule A, 48 hours with both drugs), or 24h-treatment with danusertib or volasertib followed by 24h-treatment with danusertib or vo-lasertib+MK1775 (schedule B). No relevant toxic effects were observed in normal cells using the drugs combination in both schedules.

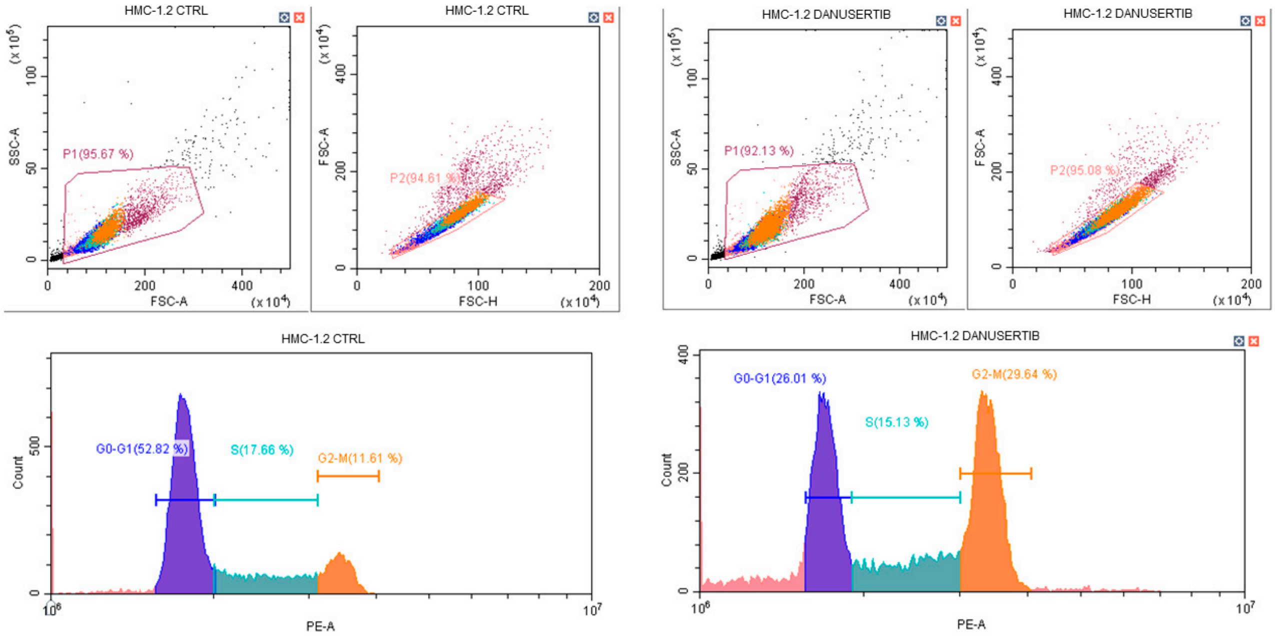

Figure S4. A representative cell cycle graph is shown below. It indicates a flow cytometry evaluation of cell cycle distri-bution of HMC-1.2 cell line untreated and treated with 100 nM danusertib for 24 hours. Cell cycle analysis was performed excluding the sub-G1 population, including dead cells.
